# Prognostic impact of hyperreflective foci in nonsyndromic retinitis pigmentosa

**DOI:** 10.1007/s00417-024-06474-1

**Published:** 2024-04-05

**Authors:** Raquel Félix, Nuno Gouveia, João Bernardes, Rufino Silva, Joaquim Murta, João Pedro Marques

**Affiliations:** 1 Centro Hospitalar e Universitário de Coimbra (CHUC), Coimbra, Portugal; 2 Clinical Academic Center of Coimbra (CACC), Coimbra, Portugal; 3 Faculty of Medicine, University of Coimbra (FMUC), Coimbra, Portugal

**Keywords:** Retinitis pigmentosa, Optical coherence tomography, Hyperreflective foci, Prognosis, Biomarkers

## Abstract

**Purpose:**

To evaluate the prognostic impact of hyperreflective foci (HRF) on spectral-domain optical coherence tomography (SD-OCT) in nonsyndromic retinitis pigmentosa (RP).

**Methods:**

Retrospective, single-center cohort study including genetically-tested RP patients with a minimum follow-up of 24 months. Clinical data including demographics, genetic results and best-corrected visual acuity (BCVA) at baseline and follow-up were collected. Horizontal and vertical SD-OCT scans were analyzed by 2 independent graders. Outer nuclear layer (ONL) thickness and ellipsoid zone (EZ) width were manually measured in horizontal and vertical scans. HRF were classified according to location: outer retinal layers within the central 3mm (central-HRF), outer retinal layers beyond the central 3mm (perifoveal-HRF), and choroid (choroidal-HRF). Central macular thickness (CMT), central point thickness (CPT) and choroidal thickness (CT) at baseline and follow-up were also recorded.

**Results:**

A total of 175 eyes from 94 RP patients (47.9% female, mean age 50.7±15.5 years) were included, with a mean follow-up of 29.24±7.17 months. Mean ETDRS (early treatment diabetic retinopathy study) BCVA decreased from 61.09±23.54 to 56.09±26.65 (*p*=0.082). At baseline, 72 eyes (41.1%) showed central-HRF, 110 eyes (62.9%) had perifoveal-HRF and 149 eyes (85.1%) exhibited choroidal-HRF. Central-HRF and perifoveal-HRF were associated with worse final BCVA, as well as greater BCVA deterioration (all *p*<0.0029). Only central-HRF were associated with a worse final CMT (*p*<0.001). Shorter EZ widths were associated with all types of HRF (*p*<0.05). Perifoveal and choroidal-HRF predicted smaller final EZ areas (*p*<0.01).

**Conclusion:**

HRF are highly prevalent in RP patients and appear to have a negative prognostic impact in visual function and EZ area.

## Key messages


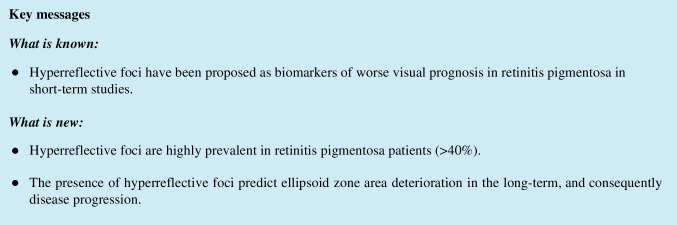


## Introduction

Retinitis pigmentosa (RP) is the most common form of inherited retinal disease (IRD) and represents a major cause of visual disability, with an estimated prevalence of 1:4000 and more than 1.5 million patients affected worldwide [1].

RP comprises a group of progressive IRDs characterized by progressive degeneration of photoreceptors and retinal pigment epithelium (RPE) [1]. Primary degeneration of rod photoreceptors causes the initial symptoms of nyctalopia and difficulty with dark adaptation, followed by progressive loss of the visual field in a concentric pattern. Cone photoreceptor cell death occurs at later stages, leading to a gradual decrease in central vision [1]. The classical triad of RP clinical features includes bone spicule pigmentation, attenuation of retinal vessels, and a waxy pallor of the optic nerve [1].

Disease progression can be monitored based on visual acuity, visual field testing, fundus autofluorescence and optical coherence tomography (OCT) [2]. OCT biomarkers such as outer retinal layer integrity and ellipsoid zone (EZ) width have been widely accepted as predictors of long-term visual loss in RP [3, 4].

Hyperreflective foci (HRF) are small dense particles with high brightness on OCT imaging which may be present in the retina or choroid [5]. HRF have been described in several retinal diseases, including age-related macular degeneration, diabetic retinopathy, Stargardt disease and recently RP [3, 5–9]. Although HRF have been proposed as biomarkers of disease progression and worse visual prognosis, their pathogenic role in RP remains undetermined as long-term studies are currently lacking [3, 5, 6].

The purpose of this study was to evaluate the association between the presence of HRF on spectral-domain OCT (SD-OCT) and RP severity and progression over a minimum follow-up of 24 months.

## Methods

### Study design and population

Retrospective, observational study conducted at an IRD referral center in Portugal. Genetically-tested nonsyndromic RP patients with a minimum follow-up of 24 months were identified using the IRD-PT registry (www.retina.com.pt) [10]. All patients provided informed consent. The study was approved by the local ethics committee and followed the tenets of the Declaration of Helsinki for biomedical research.

### Clinical/demographic features

Clinical data including demographics (age and gender), genetic testing results and early treatment diabetic retinopathy study (ETDRS) best-corrected visual acuity (BCVA) at baseline and follow-up, and follow-up time were collected from each patient’s medical records. Eyes with coexisting conditions that were not inherent to the natural history of the disease were excluded from the analysis. We also excluded eyes with marked subfoveal cystoid macular edema, retinoschisis, low-quality images due do media opacities or poor eye fixation and eyes submitted to treatment with *voretigene neparvovec*. Changes in BCVA of up to 5 ETDRS letters were considered inter-visit variability. Eyes that gained more than 5 letters between baseline and the last available follow-up, due to cataract surgery or capsulotomy for posterior capsule opacification, were excluded from the BCVA analyses to minimize bias.

### Spectral-domain optical coherence tomography (SD-OCT)

**Fig. 1:**
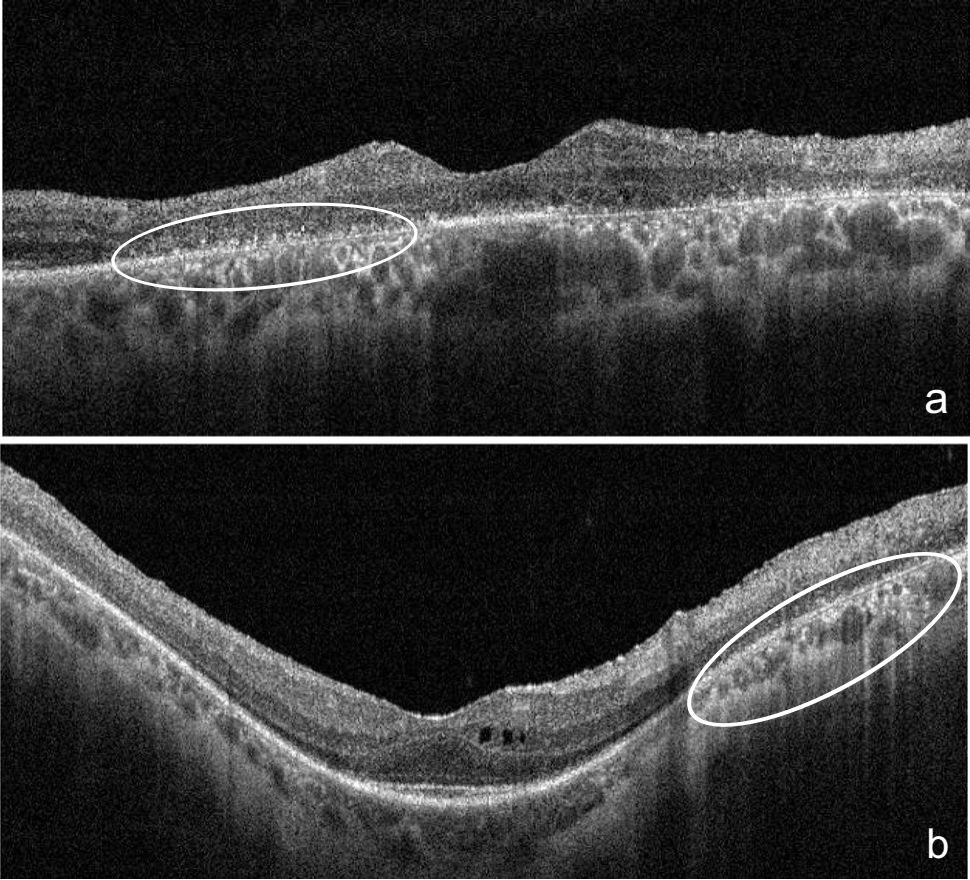
Spectral-domain optical coherence tomography scans of retinitis pigmentosa patients, showing hyperreflective foci in the outer retinal layers (**a**) and choroid (**b**)

All patients underwent SD-OCT imaging (Avanti RTVue XR 100, Optovue Inc, Fremont, CA, USA) at baseline and follow-up (minimum 24 months). Horizontal and vertical SD-OCT scans were blindly analyzed (irrespective of patient demographics, genetic testing results, or visual acuity) by 2 independent certified medical graders (RF and NG). HRF were defined as discrete, well-circumscribed hyperreflective lesions (reflectivity at least as bright as the RPE band), with a maximum size of 50μm (Fig. 1) [11]. They were identified and classified according to location on SD-OCT: (1) outer retinal layers within the central 3mm diameter (central-HRF) of the macula (corresponding to the inner ring of ETDRS grid); (2) outer retinal layers beyond the central 3mm (perifoveal-HRF); and (3) choroid (choroidal-HRF) [3].

Outer nuclear layer (ONL) thickness and ellipsoid zone (EZ) width were manually measured in horizontal and vertical scans. By assuming that the OCT EZ area is a semi-oval structure, each of the vertical and horizontal EZ widths were considered a diameter. Therefore, OCT EZ area was calculated using the following formula: EZ area $=\pi {\left(\frac{\left(\mathcal{D}1+\mathcal{D}2\right)}{4}\right)}^2$ . Central macular thickness (CMT), central point thickness (CPT) and subfoveal choroidal thickness (CT) at baseline and follow-up were also recorded.

### Statistical analysis

Variables of interest were compared based on the presence/absence of each type of HRF (central, perifoveal or choroidal) using Student’s t-test or Mann-Whitney test, according to the data’s normality status. Comparisons of mean age, final BCVA and change in BCVA between eyes with different types of HRF present were performed using Kruskal-Wallis test.

In order to test which features were predictive of RP severity and progression, we built mixed effects regression models, which adjusted for intra patient correlation (since both eyes from the same patient were included whenever possible). For model building, we first performed a univariate mixed effects analysis for each predictor. All variables with significant p-values were then included in the multivariate analysis, to control for possible confounders. The most optimal linear mixed model with the lowest quasi-likelihood information criterion was selected.

P-values less than 0.05 were considered statistically significant. Multiple test corrections according to Bonferroni were done when appropriate. All statistical analyses were conducted using SPSS software (version 23.0).

## Results

A total of 175 eyes from 94 RP patients (47.9% female, mean age 50.7±15.5 years) were included (Table 1). Mean follow-up time was 29.24±7.17 months (range 24-60 months). Mean ETDRS BCVA decreased from 61.09 ± 23.54 letters at baseline to 56.09 ± 26.65 letters at the last follow-up, corresponding to an average loss of 5 letters (*p*=0.082). A decrease of more than 5 ETDRS letters occurred in 110 eyes.

**Table 1:** Clinical and demographic characteristics and genetic testing results

Characteristic	Result
Female, *n* (%)	45 (47.9%)
Age, mean (SD)	50.67 (15.49)
Follow-up time (months), mean (SD)	29.24 (7.17)
Baseline BCVA, mean (SD)	61.09 (23.54)
Final BCVA, mean (SD)	56.09 (26.65)
HRF, *n* (%)	
Central-HRF	72 (41.1%)
Perifoveal-HRF	110 (62.9%)
Choroid-HRF	149 (85.1%)
Unsolved, *n* (%)	20 (21.3%)
Inheritance pattern, *n* (%)	
AR	58 (78.4%)
AD	6 (8.1%)
XL	10 (13.5%)
Gene, n (%)	
*EYS*	24 (32.4%)
*RPGR*	10 (13.5%)
*IMPG2*	7 (9.5%)
*CNGB1*	5 (6.8%)
*USH2A*	3 (4.1%)
*PDE6B*	3 (4.1%)
*RPE65*	2 (2.7%)

*AD* autosomal dominant, *AR* autosomal recessive, *BCVA* best-corrected visual acuity, *HRF* hyperreflective foci, *SD* standard deviation, *XL* X-linked

Genetic characterization of the sample is also presented in Table 1.

At baseline, 72 eyes (41.1%) showed central-HRF, 110 eyes (62.9%) had perifoveal-HRF and 149 eyes (85.1%) exhibited choroidal-HRF. Only 9.8% of eyes showed no HRF on OCT, while 40.5% exhibited all 3 categories of HRF. The associations between different HRF groups and other analyzed variables are presented in Table 2. When comparing the group of patients that had no HRF to those that had at least one type of HRF, we found that there were no differences in the BCVA at baseline (*p*=0.171) or follow-up (*p*=0.077), or in its variation (*p*=0.097). However there were significant differences in EZ area for these two groups, with patients that had no HRF showing better EZ areas at baseline (*p*<0.001) and follow-up (*p*=0.001).

**Table 2:** Association between HRF categories and clinical and SD-OCT features

Mean (SD)	Central-HRF			Perifoveal-HRF			Choroid-HRF		
	Absent (*n*=103)	Present (*n*=72)	*p*	Absent *n*=65	Present *n*=110	*p*	Absent *n*=26	Present *n*=149	*p*
Baseline BCVA (ETDRS letters)	67.5 (21.9)	53.3 (23.3)	**<0.001**	65.5 (24.2)	58.7 (22.9)	0.012	58.0 (29.4)	61.7 (22.4)	0.913
Final BCVA (ETDRS letters)	64.7 (24.2)	45.6 (25.9)	**<0.001**	64.1 (25)	51.7 (26.6)	**0.001**	58.0 (29.4)	55.9 (26.4)	0.640
BCVA variation (ETDRS letters)	-2.8 (8.9)	-7.7 (11.3)	**0.002**	-1.4 (6.2)	-7.0 (11.5)	**<0.001**	-1.0 (3.9)	-5.7 (10.9)	0.059
Final CMT (μm)	236.4 (59.5)	202.4 (44.8)	**<0.001**	236.5 (57.1)	214.3 (54.6)	0.013	246.5 (69.5)	218.5 (53.1)	0.024
CMT variation (μm)	-1.2 (13.7)	-8.4 (21.4)	0.009	-2.2 (11.8)	-5.3 (20.2)	0.268	-2.4 (17.6)	-4.4 (17.6)	0.596
Final CPT (μm)	197.7 (57.2)	157.8 (54.2)	**<0.001**	198.8 (57.1)	170.7 (58.2)	**0.002**	203.7 (65.8)	177.2 (57.4)	0.042
CPT variation (μm)	-4.9 (15.8)	-12.1 (39.5)	0.099	-4.4 (15.5)	-9.9 (33.6)	0.216	-9.0 (23.2)	-7.7 (29.2)	0.838
Final horizontal ONL (μm)	83.3 (33.7)	65.5 (23.1)	**<0.001**	80.8 (33.5)	73.1 (29.2)	0.112	83.8 (42.3)	74.6 (28.5)	0.161
Horizontal ONL variation (μm)	-2.1 (11.7)	-3.4 (16.1)	0.543	-3.3 (10.9)	-2.2 (15.1)	0.591	-2.9 (14.8)	-2.5 (13.5)	0.898
Final vertical ONL (μm)	84.6 (36.6)	67.2 (22.4)	**<0.001**	84.6 (38.7)	73.3 (27.9)	0.028	88.6 (53.4)	75.4 (27.2)	0.058
Vertical ONL variation (μm)	-3.6 (13.3)	-8.0 (12.0)	0.028	-2.0 (13.4)	-7.3 (12.2)	0.008	-3.4 (20.0)	-5.7 (11.2)	0.393
Final horizontal EZ (μm)	1981.0 (2311.7)	622.8 (1107.2)	**<0.001**	2265.6 (2657.3)	923.8 (1302.1)	**<0.001**	3048.0 (3530.9)	1138.5 (1465.6)	0.024
Horizontal EZ variation (μm)	-198.6 (240.9)	-145.3 (298.1)	0.042	-204.3 (254.4)	-160.4 (273.2)	0.178	-176.8 (248.2)	-176.5 (270.3)	0.714
Final vertical EZ (μm)	1946.23 (2582.0)	499.2 (927.8)	**<0.001**	2321.6 (3048.2)	780.7 (1139.1)	**<0.001**	3203.7 (3955.6)	1021.4 (1490.0)	0.049
Vertical EZ variation (μm)	-157.2 (304.5)	-131.8 (198.0)	0.304	-148.6 (324.2)	-145.6 (226.2)	0.294	-181.2 (230.7)	-140.8 (271.1)	0.734
Final CT (μm)	233.1 (100.8)	236.5 (104.8)	0.831	245.2 (91.8)	228.2 (107.8)	0.293	195.5 (118.1)	241.4 (98.0)	0.034
CT variation (μm)	4.5 (43.6)	-0.2 (41.8)	0.472	2.3 (36.7)	2.7 (46.2)	0.957	3.4 (48.0)	2.4 (42.0)	0.920

Significance level was set at *p*<0.0029 following Bonferroni correction; statistically significant findings are represented in bold. *BCVA* best-corrected visual acuity, *CMT* central macular thickness, *CPT* central point thickness, *CT* subfoveal choroidal thickness, *ETDRS* early treatment diabetic retinopathy study, *EZ* ellipsoid zone width, *HRF* hyperreflective foci, *ONL* outer nuclear layer thickness, *SD* standard deviation, *SD-OCT* spectral-domain optical coherence tomography

Central-HRF and perifoveal-HRF were associated with worse follow-up BCVA, as well as greater BCVA deterioration (all *p*<0.0029), while choroidal-HRF showed no significant associations with BCVA. Only central-HRF were associated with a worse final CMT (*p*<0.001). Smaller EZ widths at follow-up, vertically and horizontally, were associated with the presence of central and perifoveal HRF (all *p*<0.001).

We found an association between the coexistence of a larger number of HRF locations and worse final BCVA as well as a greater BCVA decline (p≤0.001) (Table 3). Age and inheritance pattern were not associated with the presence of HRF (*p*>0.1).

**Table 3:** Association between age, final BCVA and BCVA variation and the number of coexisting categories of HRF present in each eye

	Number of HRF types				*p*
	0 (*n*=15)	1 (*n*=40)	2 (*n*=36)	3 (*n*=62)	
Age (years), mean (SD)	40.73 (20.95)	32.25 (13.41)	34.58 (16.26)	38.45 (15.37)	0.185
Final BCVA (ETDRS letters), mean (SD)	67.33 (21.88)	63.15 (27.39)	59.11 (24.57)	47.06 (26.11)	**<0.001**
BCVA variation (ETDRS letters), mean (SD)	-0.87 (4.17)	-1.25 (5.77)	-5.31 (12.11)	-8.24 (11.45)	**0.001**

*BCVA* best-corrected visual acuity, *ETDRS* early treatment diabetic retinopathy study, *HRF* hyperreflective foci

Univariate and multivariate mixed effects analysis of factors associated with final BCVA are presented in Table 4. In the multivariate analysis, baseline BCVA, central macular thickness, choroidal thickness and presence of central-HRF significantly predicted final BCVA.

**Table 4:** Univariate and multivariate mixed effects analysis of factors associated with final BCVA

	β	*p*
*Univariate analysis*		
Age	0.03	0.824
Central-HRF	-19.15	**<0.001**
Perifoveal-HRF	-12.39	**0.005**
Choroid-HRF	-1.07	0.859
CMT	0.22	**<0.001**
CPT	0.23	**<0.001**
CT	0.07	**0.002**
EZ area	0.000003	**<0.001**
Mean ONL	0.38	**<0.001**
*Multivariate analysis*		
Baseline BCVA	0.97	**<0.001**
Central-HRF	-4.29	**0.018**
CMT	0.07	**0.015**
CT	0.02	**0.007**
Mean ONL	-0.08	0.114

*BCVA* best-corrected visual acuity, *CMT* central macular thickness, *CPT* central point thickness, *CT* subfoveal choroidal thickness, *EZ* ellipsoid zone, *HRF* hyperreflective foci, *ONL* outer nuclear layer thickness

Since overall variation in BCVA was not significant, the analyses of variables associated with change in BCVA were performed only in the group of eyes which had a decrease >5 ETDRS letters (*n*=110) (Table 5). In the multivariate analysis, baseline BCVA, EZ area, central macular thickness, choroidal thickness and presence of central and perifoveal-HRF were significant predictors of BCVA variation.

**Table 5:** Univariate and multivariate mixed effects analysis of factors associated with BCVA variation

	β	p
*Univariate analysis*		
Baseline BCVA	12.52	**0.007**
Central-HRF	-20.20	**<0.001**
Perifoveal-HRF	-15.36	**0.005**
Choroid-HRF	-21.30	**0.004**
CMT	0.23	**<0.001**
CPT	0.22	**<0.001**
CT	0.06	**0.007**
Mean ONL	0.40	**<0.001**
EZ area	0.000001	**<0.001**
*Multivariate analysis*		
Baseline BCVA	12.42	**<0.001**
Central-HRF	-16.54	**<0.001**
Perifoveal-HRF	-18.38	**0.002**
CMT	0.29	**<0.001**
CT	0.05	**0.006**
Mean ONL	-0.22	0.063
EZ area	0.00	**0.008**

*BCVA* best-corrected visual acuity, *CMT* central macular thickness, *CPT* central point thickness, *CT* subfoveal choroidal thickness, *EZ* ellipsoid zone, *HRF* hyperreflective foci, *ONL* outer nuclear layer thickness

In the univariate mixed models analysis, all categories of HRF were associated with smaller final EZ areas, with perifoveal and choroid-HRF showing a significant negative impact in the multivariate analysis (Table 6).

**Table 6:** Univariate and multivariate mixed effects analysis of factors associated with final EZ area

	β	p
*Univariate analysis*		
Central-HRF	-7459614.9	**0.001**
Perifoveal-HRF	-9702762.8	**<0.001**
Choroid-HRF	-18092771.9	**<0.001**
*Multivariate analysis*		
Perifoveal-HRF	-6096258.4	**0.003**
Choroid-HRF	-15600435.7	**<0.001**

*HRF* hyperreflective foci, *EZ* ellipsoid zone

## Discussion

SD-OCT provides high-resolution visualization of the cross-sectional morphology of the retina and can greatly contribute to monitor the severity and progression of RP [2, 12, 13]. Disruption of the EZ, thickness of the ONL, and integrity of the ELM have been proposed as SD-OCT biomarkers indicating photoreceptor degeneration in RP [3, 13].

In this study, we found a high prevalence of HRF in RP patients, with >40% of patients exhibiting coexistence of the 3 different categories of HRF on SD-OCT. The origin of HRF in retinal diseases has been widely discussed. It has been hypothesized that HRF may represent lipid extravasation, microglial proliferation in damaged retina or migrating RPE cells [3, 5, 6]. A histopathologic study found that RPE cells migrate to neurosensory retina in response to photoreceptor degeneration [14]. The most likely hypothesis in RP is that HRF in outer retinal layers originate from photoreceptor cell death, with subsequent RPE cell degeneration and migration into the ONL, possibly as a reparative response [3, 6]. In accordance with this hypothesis, we found a higher proportion of eyes without HRF in the central area in comparison to extrafoveal locations. It is our belief that this happens because central foveal cell degeneration and atrophy occur in later disease stages, and only in more severe cases. Nagasaka et al [5] found a positive association between the outer retinal HRF and aqueous flare values, suggesting that HRF may also reflect the severity of RP-associated intraocular inflammation. Additionally, HRF have been found in the choroid, and have been shown to be more prominent in areas of atrophic retina, which may be explained by the lower blockage of OCT signal in these areas, possibly unmasking choroidal melanocyte as hyperreflective spots [3]. Huang et al [3] demonstrated spatial relationships between HRF and disrupted photoreceptor areas, suggesting that RPE cells migrate to regions of photoreceptor degeneration. This is also supported by the finding of an overlap of areas of low auto-fluorescence with regions of high HRF numbers [5]. Low auto-fluorescence areas represent defects of the RPE layer, which have been postulated to occur due to RPE cell migration in response to photoreceptor degeneration, as mentioned previously. This migration in turn leads to decreased reflection of the RPE layer, which may manifest as HRF on OCT, as well as areas of low auto-fluorescence on fundus auto-fluorescence [3].

In our study, only central and perifoveal-HRF demonstrated prognostic impact on visual acuity, with choroidal foci showing no significant associations with BCVA. This differs from a previous study in which all 3 types of HRF showed an association with visual acuity [3]. One study also found an association between outer retinal HRF and visual field loss in RP, reflecting the extent of photoreceptor degeneration and subsequent disease severity [5].

In our population, all locations of HRF were associated with decreased macular thickness. This is in accordance to previous studies, which revealed significant associations between HRF and retinal thinning [3, 5]. We also found an association between the coexistence of a larger number of HRF locations and worse visual function, which is in line with previous studies. The number of HRF in the macular region of patients with other retinal diseases, such as age-related macular degeneration and Stargardt disease has been negatively associated with visual function [15, 16]. In RP, an accumulating effect of the presence of HRF, with worse visual acuity in patients with 2 or 3 types of HRF has also been described [3].

Visual acuity is the primary marker of visual function in clinical and research settings [2]. However, since the central retina remains relatively preserved until the final stages of RP, BCVA may not always be an accurate measure of disease progression, particularly in earlier stages of the disease [1]. Although several studies have revealed an association between visual acuity in RP patients and the condition of the EZ, this may also be an important marker of disease progression in patients whose central visual acuity has not yet been compromised, since it correlates to visual field boundaries and has also been associated with a decrease in visual field sensitivity [1, 17]. Previous studies reported significant progression rate in EZ width over time, with the rate of decline in EZ being consistent with those for visual fields, therefore verifying the utility of these measurements for disease monitoring purposes [17, 18]. The integrity and extent of the EZ has been found to strongly correlate retinal structure with function, and in particular self-reported visual function and disability, making it an important structural biomarker and outcome measure in therapeutic trials [2, 4]. We found that patients with no HRF showed better EZ areas at baseline and follow-up, and lower loss of EZ area over time, when compared to patients that showed at least one type of HRF. Moreover, in our study, all three types of HRF showed a significant negative prognostic impact on EZ area, with perifoveal and choroid-HRF exhibiting a strong and independently significant impact on final EZ area, observed on multivariate analysis. EZ area calculation was carried out since it has been proposed that using EZ area instead of its width may be an improvement for evaluating progression on SD-OCT [4, 19]. Although both variables are highly correlated, EZ area provides a more comprehensive assessment of the total EZ, therefore enabling better structure-function correlations since it can be more easily compared with visual field testing [4, 19]. This result may be of particular importance for longitudinal evaluations if rates of EZ changes in different meridians vary [19].

Limitations of this study include its retrospective nature, with an inherent heterogeneity of follow-up visits. Additionally, for SD-OCT measurements, the macular curvature was not accounted for, which may have led to an underestimation of EZ width. Nevertheless, by thoroughly analyzing a large patient cohort for a minimum follow-up of 24 months, this study provides strong evidence about the prevalence and negative prognostic impact of HRF in nonsyndromic RP.

In conclusion, HRF are highly prevalent in nonsyndromic RP patients and were shown to have a negative prognostic impact in visual function and EZ area. Central macular thickness and choroidal thickness were also found to be predictors of visual prognosis.

## Declarations

This work was presented at the 47th Brazillian Retina and Vitreous Society (BRAVS) meeting where it was awarded the Marcos Ávila Award (Best BRAVS Free Paper) and Walter Takahashi Award (Best Clinical Free Paper).
